# Efficacy and utility of a tool for both measurement of vitamin B6, B12, D, folate and iron status and assessment of diet quality in athletes

**DOI:** 10.1017/jns.2023.68

**Published:** 2023-07-17

**Authors:** A. Mireille Baart, Anne I. Slotegraaf, Inge E. Gobes-de Punder, Marco Mensink, Floris Wardenaar, Jeanne H.M. de Vries, Jacqueline M.T. Klein Gunnewiek, Michiel G.J. Balvers, Rieneke Terink

**Affiliations:** 1 Sports Valley, Department of Sports Medicine, Gelderse Vallei Hospital, Ede, The Netherlands; 2 Division of Human Nutrition and Health, Wageningen University & Research, Wageningen, The Netherlands; 3 Clinical Chemistry and Haematology Laboratory, Gelderse Vallei Hospital, Ede, The Netherlands; 4 Nutrition and Healthcare Alliance, Ede, The Netherlands; 5 College of Health Solutions, Arizona State University, Phoenix, AZ, USA

**Keywords:** Athletes, Diet quality, Micronutrient status, Screener

## Abstract

NutriProfiel® is a tool to measure micronutrient status and to assess diet quality. It consists of measurement of micronutrient status in blood and a short food frequency questionnaire (FFQ) (‘Eetscore-FFQ’). Based on the results, individuals receive a dietary advice. In this study, we evaluated the application of NutriProfiel in athletes (‘NutriProfiel-Sport’) by assessing the coverage of nutrient intake of the Eetscore-FFQ (part 1) and by evaluating athlete's dietary behaviour after using NutriProfiel-Sport and their satisfaction with this tool (part 2). For part 1, data of 419 athletes were used. We evaluated the coverage of nutrient intake of the Eetscore-FFQ using first and second MOMents (MOM1 and MOM2) sum scores of food items in the questionnaire. Forty-eight athletes were involved in part 2. They gave blood samples for micronutrient status measurement and were asked to complete the Eetscore-FFQ at baseline and after 3 months, as well as a questionnaire on their satisfaction with NutriProfiel-Sport. Results showed that for most nutrients, MOM1 and MOM2 scores were above 80 %, meaning that nutrient intake was sufficiently covered by the Eetscore-FFQ. No difference in diet quality was observed between baseline and after 3 months. Nevertheless, a majority of athletes reported the NutriProfiel-Sport results and advice as useful. On a scale from 0 to 10, NutriProfiel-Sport was graded with a mean (±sd) score of 7⋅6 (±0⋅8). In conclusion, NutriProfiel-Sport is a potential valuable and appreciated tool for athletes and the Eetscore-FFQ as part of this tool sufficiently covers nutrient intake in athletes.

## Introduction

Micronutrients, including vitamins and minerals, are essential for health and performance. They play important roles in energy metabolism, growth, bone health, immune function, blood clotting, fluid balance and several other processes^(1)^. A deficiency in micronutrients can cause serious health problems^(2)^ and may impair performance in athletes^(2)^. Athletes can be at risk for micronutrient deficiencies because of inadequate intake and/or increased losses. Especially in sports where a low body weight is thought to be an advantage, for example in gymnastics, or weight-class sports such as judo or rowing, but also in endurance sports were a high power-to-mass ratio is an advantage, inadequate micronutrient intake frequently occurs^(3)^. Increased loss of micronutrients during exercise may also occur, for example because of losses in sweat or through increased haemolysis^(4)^. Although studies suggest that micronutrient supplements are frequently used by athletes^(5–7)^, micronutrient deficiencies in athletes occur, especially deficiencies of vitamin D and iron^(8–10)^. Monitoring athlete's micronutrient status aimed at optimising micronutrient intake might thus be valuable.

Micronutrient deficiencies are also present in the general population and are commonly seen in patients in our hospital. In an effort to provide better patient care, we developed NutriProfiel®. This is a web-based tool that combines measurement of micronutrient status in blood with a screening questionnaire to assess diet quality^(11)^. Based on the results, individuals receive a personalised dietary advice which is automatically generated by the NutriProfiel tool. The following parameters are measured in blood: vitamin B6, folate, vitamin B12, vitamin D, haemoglobin and ferritin. The screening questionnaire is a short food frequency questionnaire (FFQ), called the Eetscore-FFQ^(12)^, which was developed to score the Dutch Healthy Diet index 2015 (DHD2015-index)^(13)^, a quantitative measure for adherence to the Dutch dietary guidelines 2015^(14,15)^, rather than to assess absolute dietary intake. The combined assessment of both blood parameters and diet quality is important for a good interpretation of the micronutrient status. If micronutrient concentrations are insufficient and the dietary guidelines are not sufficiently met, a dietary advice could help to improve micronutrient status. If micronutrient concentrations are insufficient while the dietary guidelines are met, there might be a physiological problem and further diagnostic tests are warranted.

NutriProfiel is in use in the Gelderse Vallei Hospital, Ede, The Netherlands, since 2015, for application in patients. However, NutriProfiel may also be useful for measurement of micronutrient status and diet quality in athletes. The NutriProfiel blood parameters are of particular interest for athletes because they are needed for tissue repair after exercise as they are involved in the methylation cycle, which is required for DNA synthesis (vitamin B6, folate and vitamin B12)^(16)^, because of their contribution to erythropoiesis and oxygen transport capacity (folate, vitamin B12, haemoglobin and ferritin)^(17)^, because of their contribution to bone health and muscle strength (vitamin D)^(18)^, and because of the potential harmful effects of over supplementation (vitamin B6)^(19)^, which is observed among athletes. The Eetscore-FFQ of the tool has been shown to be a valid method to assess diet quality in the average Dutch population^(12)^. However, dietary intake of athletes may differ from the general population. Athletes may for example consume other foods, e.g. specific sports nutrition products. Thus, it is important to first assess whether the food items in the Eetscore-FFQ sufficiently cover nutrient intake in athletes before NutriProfiel can be used in athletes. Second, the efficacy and utility of the tool in an athletic population should be evaluated.

The aim of the present study is to evaluate the application of NutriProfiel in athletes, further referred to as ‘NutriProfiel-Sport’. The study consists of two parts. In part 1, the coverage of nutrient intake of the Eetscore-FFQ in athletes will be evaluated. In part 2, we will evaluate the efficacy and utility of the NutriProfiel-Sport tool. For this purpose, we will examine micronutrient status and diet quality in athletes and investigate whether diet quality improves after athletes receive a dietary advice from the NutriProfiel-Sport tool. In addition, we will evaluate the satisfaction of athletes with the NutriProfiel-Sport tool.

## Methods

### Part 1

#### Study population

For the evaluation of the Eetscore-FFQ in athletes, dietary intake data of Dutch athletes collected in a previous study was used. A comprehensive description of the study population and data collection can be found elsewhere^(20,21)^. In brief, Dutch athletes, practicing eighteen different types of sport, with a relatively high level of exercise and a minimum average exercise duration of 9 hours a week, were recruited. For the present study, athletes with incomplete dietary intake data and athletes with implausible energy intake (EI) were excluded. The latter was evaluated using the Goldberg cutoff method^(22,23)^: the reported EI divided by the estimated basal metabolic rate (BMR) according to Schofield's formula^(24)^, the EI/BMR index, was compared to a cutoff limit of 0⋅87 on an individual level.

#### Evaluation of the Eetscore-FFQ

The study from which data was used^(20,21)^ was performed between February 2012 and June 2015. Athletes were asked to complete three to four online 24-h dietary recalls, preferably during an average training phase, rather than during a rest or competition period. The 24-h dietary recalls were randomly scheduled during a 2–4 week period, on three non-consecutive days, with a minimum of 4 d and a maximum of 1 week apart. The selected days included at least two week days and one weekend day. Data were collected throughout the year. The 24-h dietary recalls were collected using ‘Compl-eat™’. This program is based on the five-step multiple-pass method, a validated technique to increase the accuracy of dietary recalls^(25,26)^. Trained dietitians checked the 24-h dietary recalls and questionnaires for completeness and unusual portion sizes. If information was missing, this was retrieved by contacting the athletes. The reported food consumption was converted into energy and nutrient intake using the Dutch Food Composition Database of 2010, the Dutch database for dietary supplements (NES) and individual food ingredient declarations. For each individual athlete, the average daily energy and nutrient intake was calculated from the multiple 24-h dietary recalls.

The Eetscore-FFQ was evaluated on coverage of macronutrients and on coverage of the micronutrients corresponding to the blood parameters that are measured with the NutriProfiel-Sport tool: vitamin B6, folate equivalents, vitamin B12, vitamin D and iron. The coverage was evaluated on the basis of two aspects. The first aspect was the contributing percentage of each separate food reported in the 24-h dietary recall to the total level of macro- and micronutrient intake. These percentages are known as the first MOMent (MOM1) of the intake of a nutrient^(27–29)^. The second aspect was the contributing percentage of each separate food reported in the 24-h dietary recall to the between-person variability in macro- and micronutrient intake. This is the second MOMent (MOM2) of the nutrient intake distribution^(29)^. The latter is important, for example, to be able to establish associations with other factors, such as in epidemiological studies. The foods that were reported in the 24-h dietary recalls were as much as possible assigned to food items of the Eetscore-FFQ. For example, the food ‘smoked salmon’ from the 24-h dietary recall was assigned to the food item ‘fatty fish’ from the Eetscore-FFQ. Next, sum scores of MOM1 and MOM2 values for each nutrient were calculated by summation of respectively MOM1 and MOM2 values of foods in the 24-h dietary recall that could be assigned to food items in the Eetscore-FFQ. Based on these sum scores, it was investigated whether nutrient intake of athletes was sufficiently covered by the Eetscore-FFQ or if addition of certain foods could improve the dietary assessment. ‘Sufficiently covered’ was defined as a sum score for MOM1 values of at least 80 %, and for MOM2 values of at least 80 % as well^(30)^. In case the sum scores of MOM1 and/or MOM2 values for a specific nutrient were below 80 %, it was investigated whether addition of foods in the 24-h dietary recall with a MOM1 and/or MOM2 value of at least 1 % not yet present in the Eetscore-FFQ could improve the assessment of nutrient intake using the Eetscore-FFQ.

Based on the results of part 1 (see Section Results), we decided not to adjust the Eetscore-FFQ for NutriProfiel-Sport.

### Part 2

#### Study design and population

**Fig. 1.**
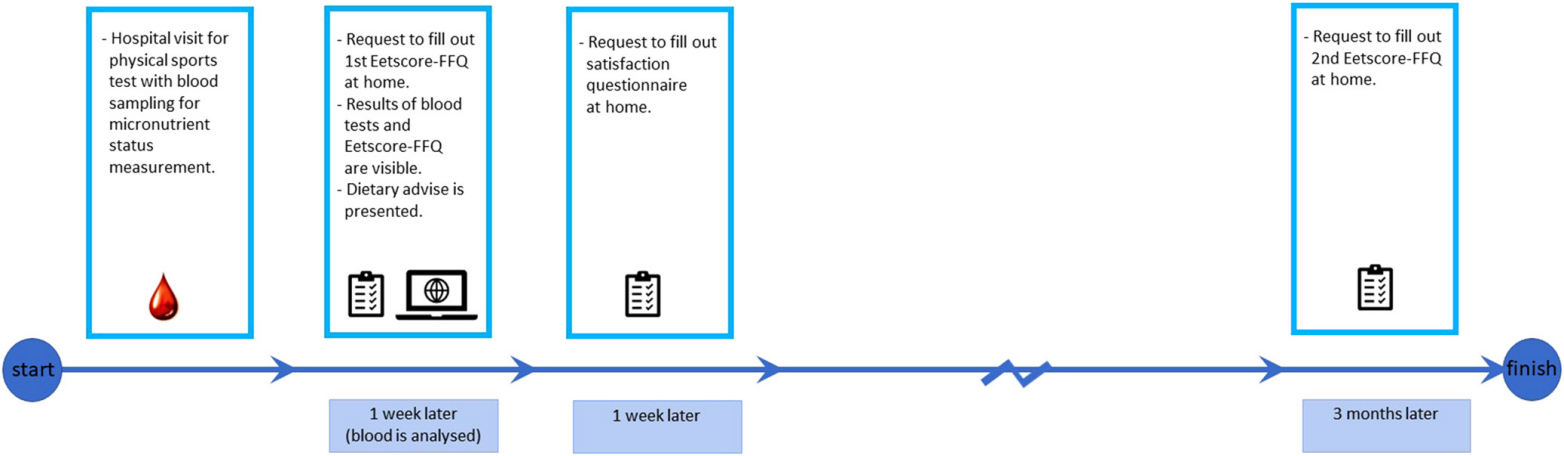
Study design for part 2.

For the evaluation of the NutriProfiel-Sport tool, athletes who visited the Sports Medicine Department of the Gelderse Vallei Hospital for a physical sports test including blood analysis in the period December 2021 to May 2022 were asked to participate in the study on the evaluation of NutriProfiel-Sport. Inclusion criteria were at least 18 years of age and be able to read and understand the Dutch language. In addition, athletes should not have dietary restrictions such as food intolerances, but following a vegetarian diet was allowed. During their visit for a physical sports test, athletes gave additional blood samples for measurement of the NutriProfiel-Sport parameters. After 1 week, when the results of the blood sample analyses were known, they received an invitation by email to fill out the online Eetscore-FFQ (baseline) of the NutriProfiel-Sport tool. Right after completing the Eetscore-FFQ, the results from both the blood tests and the Eetscore-FFQ, as well as a dietary advice based on these results, were presented to the athletes on the webpage of the NutriProfiel-Sport tool. Within 1 week, athletes received another email with an invitation to fill out an online questionnaire about their satisfaction with the tool and to grade NutriProfiel-Sport. After 3 months, athletes were asked to fill out a second online Eetscore-FFQ (follow-up). All the questionnaires could be completed at home. An overview of the study design for part 2 is presented in Fig. 1.

This study was conducted according to the guidelines laid down in the Declaration of Helsinki and approved by the Medical Ethical Committee of Utrecht University Medical Center (approval number NL7401908120). Written informed consent was obtained from all athletes prior to participation.

#### Micronutrient status measurement

The following parameters were measured in blood: vitamin B6, folate, vitamin B12, vitamin D, haemoglobin and ferritin. Venous blood samples (10 ml in total) were taken from the cephalic vein and collected in a 3 ml EDTA vacutainer for vitamin B6 analysis, in a 3⋅5 ml serum vacutainer for folate and vitamin D analysis and in a 3⋅5 ml lithium heparin gel vacutainer for vitamin B12 and ferritin analyses.

The blood sample analyses were performed in the Clinical Chemistry and Haematology Laboratory of the Gelderse Vallei Hospital by trained technicians using standard operating procedures. Whole blood vitamin B6 concentrations and serum 25(OH)D concentrations were determined with methods using liquid chromatography coupled to tandem mass spectrometry (LC-MS/MS) consisting of a Waters Acquity UPLC I-Class system coupled to a Waters Xevo TQ-S micro triple quadrupole mass spectrometer. Plasma concentrations of folate, vitamin B12 and ferritin were analysed using the Siemens Dimension Vista® System. For measurement of folate and vitamin B12, a quantitative competitive chemoluminescence assay based on LOCI® technology was used. For measurement of ferritin, a quantitative sandwich chemoluminescence assay based on LOCI® technology was used. Intra- and interassay variability of all the parameter tests have been evaluated according to the Clinical and Laboratory Standards Institute (CLSI; Wayne, PA, USA) EP10 protocol. On five consecutive days, quality control samples with three concentration levels (low–mid–high) were measured to monitor the analytical performance of the tests. All tests passed the evaluation.

Prevalence rates of concentrations below and above reference ranges were calculated according to the reference ranges used in the Gelderse Vallei Hospital^(31)^.

#### Diet quality assessment

Dietary intake was assessed with the online Eetscore-FFQ^(12)^, at baseline and at follow-up. The Eetscore-FFQ is a short FFQ, especially developed to score the DHD2015-index^(13)^, a quantitative measure for adherence to the Dutch dietary guidelines 2015^(14,15)^. It was developed using data from the Dutch National Food Consumption Survey (DNFCS) 2007–2010^(32)^. Only foods that were part of the Dutch dietary guidelines were selected in the Eetscore-FFQ. In addition, foods related to the unhealthy foods component of the DHD2015-index (see below) were selected. This resulted in a questionnaire of forty questions covering fifty-five food items, which together accounted for 85 % of EI from the adult population of the DNFCS^(12)^. Comparison of the DHD2015-index calculated from the Eetscore-FFQ and from a full-length FFQ that was validated for EI, macronutrients, dietary fibre and vitamins^(33,34)^ revealed a Kendall's tau-b correlation coefficient of 0⋅51 (95 % confidence interval (CI): 0⋅47, 0⋅55)^(12)^. The intraclass correlation coefficient between DHD2015-index scores derived from two repeated Eetscore-FFQs (with an average interval of 3⋅8 months) was 0⋅91 (95 % CI 0⋅89, 0⋅93)^(12)^.

With data obtained from the Eetscore-FFQ, the DHD2015-index was calculated. This index consists of sixteen components of which fifteen refer to the Dutch dietary guidelines 2015 from the Health Council of the Netherlands^(14,15)^: vegetables, fruits, wholegrain products, legumes, nuts, dairy, fish, tea, fats and oils, coffee, red meat, processed meat, sweetened beverages and fruit juices, alcohol and salt. The sixteenth component is on unhealthy foods^(12)^ and is based on a guideline of the Netherlands Nutrition Centre^(35)^. For all components, a score was calculated based on adherence to the guidelines. For all components, a minimum of 0 points and a maximum of 10 points can be allocated, resulting in a total score ranging from 0 to 160 points, with a higher score indicating better adherence to the guidelines. A more detailed description of the DHD2015-index can be found elsewhere^(12,13)^.

In addition to the DHD2015-index score and its component scores, scores were also calculated for intake of the NutriProfiel-Sport micronutrients and protein. The protein score was especially added for the NutriProfiel-Sport tool for athletes because adequate protein intake is particularly important for athletes. For athletes practicing resistance exercise, adequate protein intake is important for building and maintaining muscle mass, for endurance athletes this is important to offset muscle damage and promote recovery after exercise^(36)^. The micronutrient and protein scores also range from 0 to 10 points, with higher scores meaning better compliance with the recommended dietary allowance by the Health Council of the Netherlands^(37)^. An overview of the scoring per DHD2015-index component, the micronutrient scores and the protein scores is presented in Supplementary Table S1.

#### Dietary advice

The dietary advice was automatically generated by the NutriProfiel-Sport tool. It was personalised based on both the micronutrient status in blood and the calculated DHD2015-index, and was provided for each component of the DHD2015-index as well as for each micronutrient and for protein. Specific dietary suggestions for vegetarians were also included in the advice. Examples of blood tests results, a DHD2015-index score result and a personalised dietary advice are presented in Supplementary Figs. S1–S3, respectively.

#### Evaluation of athlete's satisfaction with NutriProfiel-Sport

The satisfaction of athletes with NutriProfiel-Sport was assessed with an online questionnaire, which they were asked to complete when they received their results and dietary advice. This questionnaire contained several questions on the clarity of the results and the usefulness of the advice. Athletes were also asked to grade NutriProfiel-Sport on a scale from 0 (very bad) to 10 (very good).

### Statistical analyses

Data from part 1 and part 2 were first checked for normality using a Kolmogorov–Smirnov test and visual inspection of Q-Q normality plots. Thereafter, descriptive analyses were performed. Data are presented as mean (±sd) for normal distributed variables, median (25th–75th percentile) for non-normal distributed variables, and as *n* (%) for categorical variables.

For part 2, besides descriptive analyses, the DHD2015-index total score and its component scores were compared at baseline and at follow-up. The total score was normally distributed and was compared using a paired *t*-test; the non-normally distributed component scores were compared using a Wilcoxon signed-rank test.

Statistical analyses were performed with SPSS, Version 25, SPSS, Inc., Chicago, IL.

## Results

### Part 1

#### Study population

From the 553 athletes that comprised the study population of the previous study^(20,21)^, 121 athletes were excluded because from those no complete data was available. Another thirteen athletes were excluded because they reported implausible EI, i.e. the EI/BMR index was below 0⋅87. Data from 419 athletes (248 men (59 %) and 171 women (41 %)) was used for the present study. These athletes were a representative sample from the original study population of 553 athletes and study population characteristics can be read in the previously published articles^(20,21)^.

#### Evaluation of the Eetscore-FFQ

Table 1 shows the sum scores of the MOM1 and MOM2 values of food items in the Eetscore-FFQ for macronutrients and the NutriProfiel-Sport micronutrients. For most nutrients, the MOM1 and MOM2 sum scores were at least 80 %, meaning that nutrient intake was sufficiently covered. Only for vitamin B12 and vitamin D, the MOM1 sum scores were slightly lower (76⋅2 and 79⋅6 %, respectively) and for vitamin B12, the MOM2 sum score was only 39⋅1 %.

**Table 1.** Sum scores of MOM1 and MOM2 values of food products in the Eetscore-FFQ per nutrient (*n* 419)

Nutrient	MOM1 sum score	MOM2 sum score
Total carbohydrates	84⋅0	82⋅4
Total protein	87⋅0	88⋅6
Total fat	83⋅9	84⋅7
Vitamin B6	84⋅3	90⋅0
Folate equivalents	85⋅8	90⋅9
Vitamin B12	76⋅2	39⋅1
Vitamin D	79⋅6	88⋅2
Total iron	83⋅4	87⋅1
Haem iron	95⋅1	98⋅7
Non-haem iron	82⋅3	86⋅4

The first MOMent (MOM1) values represent the contributing percentage of each separate food product in the 24-h dietary recall to the total intake of one specific nutrient. The second MOMent (MOM2) values represent the contributing percentage of each separate food in the 24-h dietary recall to the between-person variability of the intake of one specific nutrient. Sum scores of MOM1 and MOM2 values for each nutrient were calculated by summation of MOM1 and MOM2 values of food items in the Eetscore-FFQ. The coverage of nutrient intake was defined as ‘sufficiently covered’ if the sum score for MOM1 values was at least 80 %, and at least 80 % for MOM2 values as well.

To investigate whether the sum scores of MOM1 and MOM2 values for vitamin B12 and vitamin D could be increased to above 80 %, we identified foods reported in the 24-h dietary recall with MOM1 and/or MOM2 values of at least 1 % that were not yet present in the Eetscore-FFQ. These foods were eggs of any type (cooked, baked, raw, egg yolk, egg white) and shrimps. Addition of these foods to the Eetscore-FFQ would increase the MOM1 sum score for vitamin B12 and vitamin D to 93⋅1 and 90⋅4 %, respectively, and would increase the MOM2 sum score for these vitamins to 94⋅6 and 94⋅2 %, respectively.

### Part 2

#### Study population

**Fig. 2.**
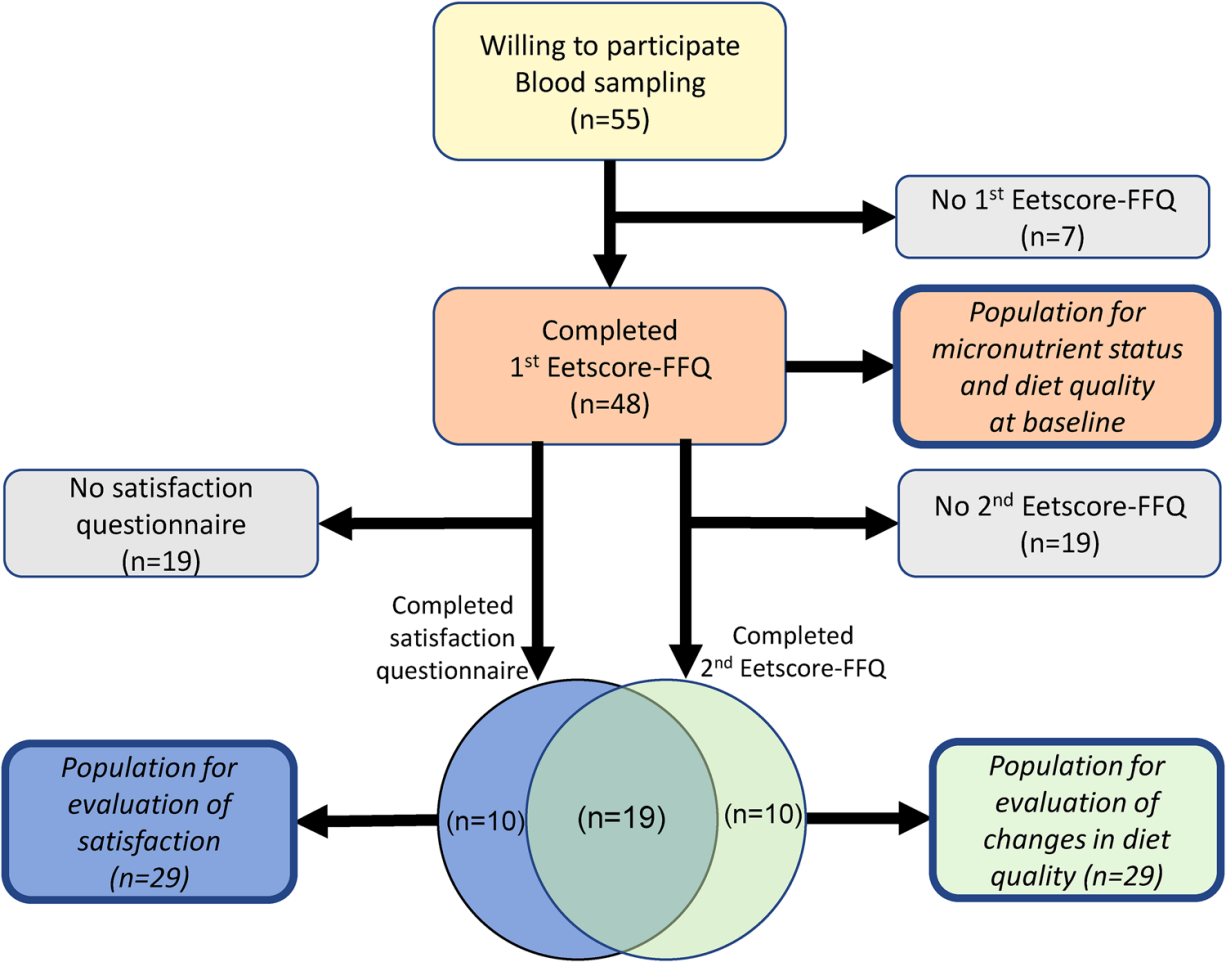
Participant flow of the study population for part 2.

A total of fifty-five athletes indicated to be willing to participate in the study on the evaluation of NutriProfiel-Sport. From these fifty-five athletes, who gave blood samples during their physical sports test, forty-eight athletes (twenty-nine men, nineteen women) completed the first Eetscore-FFQ. This group of forty-eight athletes was used to evaluate micronutrient status and diet quality at baseline. The characteristics of this group are presented in Table 2. From these forty-eight athletes, twenty-nine athletes (17 men (59 %), 12 women (41 %)) completed the second Eetscore-FFQ as well. This group was used to investigate whether the dietary advice improves diet quality. Another twenty-nine athletes (18 men (62 %), 11 women (38 %)), partly other athletes than those who completed both Eetscore-FFQs, completed the satisfaction questionnaire after they received their results from the first Eetscore-FFQ and the dietary advice. This group was used to evaluate the satisfaction of athletes with NutriProfiel-Sport. A participant flow of the study population for part 2 of this study is presented in Fig. 2.

**Table 2.** Characteristics of the study population for part 2 (*n* 48)

Characteristic	Total (*n* 48)	Men (*n* 29)	Women (*n* 19)
Age, years	31 ± 16	33 ± 17	27 ± 13
Height, m	1⋅80 ± 0⋅08	1⋅84 ± 0⋅07	1⋅74 ± 0⋅05
Weight, kg	72⋅4 ± 8⋅1	75⋅0 ± 7⋅3	68⋅5 ± 7⋅9
BMI, kg/m^2^	22⋅3 ± 2⋅5	22⋅1 ± 1⋅9	22⋅6 ± 3⋅3
Main sport			
Running	10 (21)	7 (24)	3 (16)
Cycling	10 (21)	9 (31)	1 (5)
Rowing	24 (50)	9 (31)	15 (79)
Other (boxing, fitness, triathlon, swimming)	4 (8)	4 (14)	0 (0)
Diet			
Eating meat regularly	27 (56)	21 (72)	6 (32)
Eating meat occasionally/flexitarian	7 (15)	4 (14)	3 (16)
Vegetarian	14 (29)	4 (14)	10 (53)
Supplement use			
Yes	22 (46)	13 (45)	9 (47)
No	24 (50)	14 (48)	10 (53)
Unknown	2 (4)	2 (7)	0 (0)

Data are presented as mean ± sd or as *n* (%).

#### Micronutrient status and diet quality

Micronutrient concentrations at baseline are presented in Table 3. Deficiencies were observed for vitamin D (in five out of twenty-nine men and in seven out of nineteen women) and iron, the latter reflected as low concentrations of haemoglobin (in two women) and ferritin (in seven women). From the twelve athletes with vitamin D deficiency, eight were vegetarian and one was flexitarian; from the women with low haemoglobin concentrations, one was vegetarian and one was flexitarian; from the women with low ferritin concentrations, six were vegetarian and one was flexitarian. Concentrations above reference ranges were observed for vitamin B6 (three men), folate (two men, one woman), vitamin B12 (four men) and ferritin (one man). The majority of those with high concentrations indicated to use supplements. Unfortunately, we did not exactly question which supplements were used and which dose.

**Table 3.** Micronutrient status in blood at baseline (*n* 48)

Parameter	Men (*n* 29)	Women (*n* 19)
Micronutrient concentrations		
Vitamin B6, nmol/l	114 ± 55	97 ± 19
Folate, nmol/l	24 ± 8	24 ± 7
Vitamin B12, pmol/l	415 ± 150	338 ± 60
Vitamin D, nmol/l	72 ± 27	64 ± 28
Haemoglobin, mmol/l	9⋅0 ± 0⋅4	8⋅3 ± 0⋅6
Ferritin, μg/l	62 ± 44	25 ± 20
Micronutrient concentrations related to reference ranges		
Vitamin B6		
Below reference range, <51 nmol/l	0 (0)	0 (0)
Within reference range, 51–183 nmol/l	26 (90)	19 (100)
Above reference range, >183 nmol/l	3 (10)	0 (0)
Folate		
Below reference range, <7 nmol/l	0 (0)	0 (0)
Within reference range, 7–40 nmol/l	27 (93)	18 (95)
Above reference range, >40 nmol/l	2 (7)	1 (5)
Vitamin B12 (B12)		
Below reference range, <150 pmol/l	0 (0)	0 (0)
Within reference range, 150–600 pmol	25 (86)	19 (100)
Above reference range, >600 pmol/l	4 (14)	0 (0)
Vitamin D		
Below reference range, <65 years <50 nmol/l, ≥65 years <75 nmol/l	5 (17)	7 (37)
Within reference range, <65 years 50–250 nmol/l, ≥65 years 75–250 nmol/l	24 (83)	12 (63)
Above reference range, >250 nmol/l	0 (0)	0 (0)
Haemoglobin		
Below reference range, men < 8⋅5 mmol/l, women < 7⋅5 mmol/l	0 (0)	2 (11)
Within reference range, men 8⋅5–11⋅0 mmol/l, women 7⋅5–10⋅0 mmol/l	29 (100)	17 (90)
Above reference range, men > 11⋅0 mmol/l, women > 10⋅0 mmol/l	0 (0)	0 (0)
Ferritin		
Below reference range, <15 μg/l	0 (0)	7 (37)
Within reference range, men 15–200 μg/l, women 15–150 μg/l	28 (97)	12 (63)
Above reference range, men > 200 μg/l, women > 150 μg/l	1 (3)	0 (0)

Data are presented as mean ± sd or as *n* (%).

References ranges presented here are those used in the Gelderse Vallei Hospital^(31)^.

The DHD2015-index total score, its component scores and the micronutrient and protein scores at baseline are presented in Table 4. The mean (±sd) DHD2015-index total score was 105 (±18) for men and 116 (±12) for women. There was room for improvement (i.e. the median component score was much lower than 10) for the following components: whole grain products, nuts, dairy, fish, tea processed meat, sweetened beverages and fruit juices, salt, and especially for unhealthy choices. Regarding vitamin scores, there was much room for improvement for vitamin D in both men and women, and in women also for vitamin B12, iron and protein.

**Table 4.** DHD2015-index score and its component scores at baseline (*n* 48)

Score	Men (*n* 29)	Women (*n* 19)
DHD2015-index total score^a^	105 ± 18	116 ± 12
*DHD2015-index component scores*		
1. Vegetables	10 (6–10)	9 (6–10)
2. Fruit	9 (6–9)	9 (9–10)
3. Wholegrain products	7 (5–9)	7 (6–10)
4. Legumes	10 (4–10)	10 (10–10)
5. Nuts	6 (2–10)	3 (1–7)
6. Dairy	6 (4–10)	8 (4–9)
7. Fish	6 (4–8)	3 (0–8)
8. Tea	1 (0–7)	7 (3–10)
9. Fats and oils	10 (5–10)	10 (4–10)
10. Coffee	10 (5–10)	10 (5–10)
11. Red meat	10 (10–10)	10 (10–10)
12. Processed meat	3 (0–9)	10 (8–10)
13. Sweetened beverages and fruit juices	6 (1–10)	9 (7–9)
14. Alcohol^a^	10 (10–10)	10 (10–10)
15. Salt	7 (3–9)	9 (9–10)
16. Unhealthy choices	0 (0–7)	0 (0–9)
*Micronutrient and protein scores*		
Vitamin B6^a^	10 (10–10)	9 (7–10)
Folate equivalents^a^	10 (9–10)	9 (7–10)
Vitamin B12^a^	10 (9–10)	7 (5–9)
Vitamin D^a^	3 (2–5)	1 (1–3)
Iron^a^	10 (10–10)	7 (6–8)
Protein^a^	10 (5–10)	7 (0–10)

Data are presented as mean ± sd or as median (25th–75th percentile).

a The total score is the sum of component scores 1–16. Scores for micronutrients and protein are not included in the total score.

Table 5 presents the DHD2015-index total score, its component scores and the micronutrient and protein scores at baseline and at follow-up of the twenty-nine athletes who completed both the first and the second Eetscore-FFQ. No significant differences in the total DHD2015-index scores between baseline and follow-up were observed. For men, the mean DHD2015-index total score was 105 (±21) at baseline and 109 (±20) at follow-up. For women, the total scores were 117 (±13) and 114 (±16) at baseline and follow-up, respectively. Also for most component scores and the micronutrient and protein scores, no significant differences were observed. Only the component score for legumes in men was significantly higher at follow-up than at baseline and the component score for coffee in women was significantly lower at follow-up than at baseline.

**Table 5.** DHD2015-index score and its component scores at baseline and follow-up (*n* 29)

Score	Men (*n* 17)			Women (*n* 12)		
	Baseline	Follow-up	*P*-value*	Baseline	Follow-up	*P*-value*
DHD2015-index total score^a^	105 ± 21	109 ± 20	0⋅094	117 ± 13	114 ± 16	0⋅231
*DHD2015-index component scores*						
1. Vegetables	10 (6–10)	9 (6–10)	0⋅477	10 (7–10)	10 (7–10)	0⋅176
2. Fruit	9 (5–9)	7 (5–10)	0⋅799	10 (9–10)	10 (9–10)	0⋅317
3. Wholegrain products	7 (5–9)	7 (5–10)	0⋅362	7 (5–10)	8 (6–10)	0⋅359
4. Legumes	10 (4–10)	10 (9–10)	**0⋅024**	10 (6–10)	10 (7–10)	0⋅715
5. Nuts	10 (3–10)	10 (6–10)	0⋅342	4 (1–7)	2 (1–8)	0⋅172
6. Dairy	5 (2–10)	6 (3–10)	0⋅506	7 (4–10)	6 (5–9)	0⋅754
7. Fish	6 (5–8)	8 (4–8)	0⋅592	3 (0–6)	3 (0–7)	0⋅671
8. Tea	1 (0–8)	1 (0–7)	0⋅236	6 (1–10)	6 (1–9)	0⋅726
9. Fats and oils	10 (5–10)	10 (2–10)	0⋅396	10 (3–10)	10 (0–10)	0⋅285
10. Coffee	10 (5–10)	10 (5–10)	0⋅564	10 (5–10)	5 (5–10)	**0⋅046**
11. Red meat	10 (10–10)	10 (10–10)	0⋅414	10 (10–10)	10 (10–10)	1⋅000
12. Processed meat	3 (0–9)	6 (0–10)	0⋅285	9 (8–10)	10 (10–10)	0⋅248
13. Sweetened beverages and fruit juices	8 (2–10)	9 (2–10)	0⋅091	9 (7–9)	9 (7–10)	0⋅441
14. Alcohol^a^	10 (10–10)	10 (10–10)	0⋅785	10 (10–10)	10 (10–10)	0⋅317
15. Salt	6 (3–9)	7 (5–9)	0⋅091	9 (9–10)	9 (9–9)	0⋅774
16. Unhealthy choices	0 (0–8)	0 (0–10)	0⋅285	2 (0–9)	4 (0–7)	0⋅779
*Micronutrient and protein scores*						
Vitamin B6^a^	10 (8–10)	10 (9–10)	0⋅766	10 (7–10)	9 (8–10)	0⋅959
Folate equivalents^a^	10 (9–10)	10 (9–10)	0⋅235	9 (7–10)	10 (7–10)	0⋅722
Vitamin B12^a^	10 (8–10)	10 (10–10)	0⋅314	7 (4–10)	6 (5–7)	0⋅533
Vitamin D^a^	3 (1–6)	2 (2–5)	0⋅214	1 (1–4)	2 (0–3)	0⋅959
Iron^a^	10 (10–10)	10 (10–10)	0⋅577	7 (5–8)	6 (5–9)	0⋅903
Protein^a^	10 (0–10)	10 (1–10)	1⋅000	3 (0–10)	2 (0–7)	0⋅091

Data are presented as mean ± sd or as median (25th–75th percentile).

* *P*-values for the total scores were obtained with a paired *t*-test, *P*-values for the component scores were obtained with a Wilcoxon signed-rank test; statistical significance (*P* < 0⋅05) is indicated in bold.

a The total score is the sum of component scores 1–16. Scores for micronutrients and protein are not included in the total score.

#### Evaluation of athlete's satisfaction with NutriProfiel-Sport

Table 6 presents an overview of the results obtained from the satisfaction questionnaire. All twenty-nine athletes who completed the satisfaction questionnaire reported that they read the blood tests and Eetscore results and their dietary advice. A total of twenty athletes (69 %) reported that the results from the blood tests gave them a lot of insight into their micronutrient status and eighteen athletes (62 %) reported that the results from the Eetscore gave them a lot of insight into how healthy they eat or drink. The other eleven athletes (38 %) reported that they got a little insight into their micronutrient status and/or diet quality, none of the athletes reported that they did not get any insight into this. A total of twelve athletes (41 %) knew well, sixteen athletes (55 %) knew a little and one athlete did not know at all what to do to eat or drink healthier after reviewing the dietary advice. A total of thirteen athletes (45 %) felt well motivated, fourteen athletes (48 %) felt a little motivated and two athletes (7 %) felt not motivated at all to eat or drink healthier. A total of sixteen athletes (55 %) experienced the NutriProfiel-Sport results and advice as very useful, while the other thirteen athletes (45 %) experienced it as little useful, no athlete experienced it as not useful at all. A total of eighteen athletes (62 %) experienced NutriProfiel-Sport a very valuable addition to their sport guidance, while the other eleven athletes (38 %) experienced it as a little valuable addition, no athlete experienced it as not a valuable addition at all. Regarding the Eetscore-FFQ, twenty athletes (69 %) reported that this questionnaire includes largely, and nine athletes (31 %) that it includes a little, what they eat and drink. On a scale from 0 to 10, the mean (±sd) score athletes gave to NutriProfiel-Sport was 7⋅6 (±0⋅8). A total of seven athletes reported to be willing to pay an extra amount of 10–25 euro to include NutriProfiel-Sport in their sports tests, two athletes were willing to pay an extra amount of 40 euro and another two athletes were willing to pay an extra amount of 75 euro. A total of five athletes reported not to be willing to pay extra for NutriProfiel-Sport, while thirteen athletes did not answer this question.

**Table 6.** Satisfaction of athletes with NutriProfiel-Sport (*n* 29)

	Answer (*n* %)		
Question	Yes/a lot/very/well/largely	A little	Not at all
The blood tests results gave me insight into my micronutrients status	20 (69)	9 (31)	0 (0)
The Eetscore-FFQ includes what I basically eat and drink	20 (69)	9 (31)	0 (0)
The Eetscore-FFQ lacks certain foods and drinks that I regularly consume	8 (28)	0 (0)	16 (55)
The Eetscore results gave me insight into how healthy I eat/drink	18 (62)	11 (38)	0 (0)
After reviewing my NutriProfiel-Sport advice, I knew what to do to eat/drink healthier	12 (41)	16 (55)	1 (3)
After reviewing my NutriProfiel-Sport advice, I felt motivated to eat/drink healthier	13 (45)	14 (48)	2 (7)
The NutriProfiel-Sport results and advice are practically useful	16 (55)	13 (45)	0 (0)
NutriProfiel-Sport is a valuable addition to my sport guidance	18 (62)	11 (38)	0 (0)
I Would recommend NutriProfiel-Sport to my fellow athletes	14 (48)	13 (45)	1 (3)

Data are presented as *n* (%).

## Discussion

In this study, we evaluated the application of NutriProfiel-Sport by evaluating the coverage of nutrient intake of the Eetscore-FFQ in athletes and by evaluating athlete's dietary behaviour after using NutriProfiel-Sport and their satisfaction with this tool.

The intake of macronutrients and most NutriProfiel-Sport micronutrients was sufficiently covered by the Eetscore-FFQ. Only the intake of vitamin B12 and vitamin D were less well covered.

Deficiencies, according to blood concentrations, were observed for vitamin D, haemoglobin and ferritin, while for vitamin B6, folate, vitamin B12 and ferritin, some athletes had values above the reference range. Three months after athletes had received their NutriProfiel-Sport results and advice, the quality of their diet (i.e. the DHD2015-index score) had not significantly improved. However, the majority of athletes reported that they experienced NutriProfiel-Sport as useful and as a valuable addition to their sports test and that they were either a little or very much motivated to eat and drink healthier after reviewing their NutriProfiel-Sport advice.

Intake of macronutrients and most NutriProfiel-Sport micronutrients were sufficiently covered by the Eetscore-FFQ in our population of athletes, however, intake of vitamin B12 and vitamin D was not. Addition of eggs and shrimps to the Eetscore-FFQ would increase the MOM1 and MOM2 sum scores for vitamin B12 and vitamin D to far above 80 %. The increase of these scores by the addition of shrimps can be explained by an extremely high shrimp consumption of one athlete, which can be considered as an exception. Although the addition of eggs would indeed improve the coverage of nutrient intake, we decided not to add this food item to the Eetscore-FFQ at this moment because of practical and technical reasons. One Eetscore-FFQ that can be used for patients as well as for athletes in our hospital was considered preferable. Another reason is that the Eetscore-FFQ is used as a screening tool and as such we have to make a trade-off between validity and invasiveness, i.e. the length of and required time to complete the Eetscore-FFQ. However, in future research and applications, we would recommend to add eggs to the Eetscore-FFQ.

Deficiencies of vitamin D and iron were observed in a number of athletes. Vitamin D deficiency and iron deficiency, the latter reflected as low haemoglobin and low ferritin concentrations, are quite common in athletes^(9,38)^. Vitamin D deficiency occurs more frequently in winter^(9)^, which was the period that micronutrient status was measured in the present study. Vitamin D deficiency was observed in both men and women, while low levels of haemoglobin and ferritin were observed in women only. An explanation for this observation is menstrual blood loss in women, which is the most common cause of iron deficiency anaemia^(39)^. In addition, most women with low haemoglobin and/or low ferritin concentrations were vegetarian or flexitarian, and following a vegetarian diet is known to increase the risk of iron deficiency^(40)^. Besides deficiencies, we also observed concentrations above reference ranges in a few athletes. This was the case for vitamin B6, folate, vitamin B12 and ferritin. Nutritional supplements are often used by athletes^(5,7)^, and this could explain the observed concentrations above reference ranges. Indeed, most of the athletes with concentrations above the reference ranges used supplements (but not all), however the type and dose were often unknown and it is therefore impossible to conclude whether supplement use is the cause of the high concentrations. Both the observed deficiencies and the concentrations above reference ranges show that monitoring micronutrient status in athletes is important. Especially while both deficiencies as well as high concentrations (vitamin B6) might be harmful.

Diet quality was rather high in our population of athletes. Mean DHD2015-index total scores were 105 for men and 116 for women. These scores, however, were not significantly different 3 months after athletes had received the NutriProfiel-Sport results and advice. An already high diet quality at baseline could underlie the lack of change over time. In a population of inflammatory bowel disease (IBS) patients, the mean DHD2015-index score increased significantly from 98 to 107 four months after receiving a personalised dietary advice based on the results from the Eetscore-FFQ follow-up^(41)^. This follow-up score was comparable to the baseline score for men in our population of athletes, while women in our population had an even higher score at baseline, which indicates that there was less room for improvement in our population. It could be that the diet quality of athletes with a lower diet quality would improve after receiving a NutriProfiel-Sport dietary advice.

Although diet quality did not improve in our population after athletes received the dietary advice, roughly half of the athletes indicated to be motivated to eat and drink healthier after receiving the advice. Moreover, a majority of the athletes reported the NutriProfiel-Sport results and advice as useful and valuable, and indicated that they were willing to pay a (small) extra amount to include NutriProfiel-Sport in their sports tests.

### Practical application

Results of this study show that NutriProfiel-Sport is a potential valuable and appreciated tool for measurement of vitamin B6, B12, D, folate and iron status and for assessment of diet quality in athletes. The combined assessment of both micronutrient status and diet quality is important for a good interpretation of the micronutrient status and therefore this combination is an advantage of the tool. The burden for both athletes and medical staff is low, because only one blood donation at the hospital is required, the Eetscore-FFQ can be filled out at home, and the dietary advice is automatically provided. Only in case of deficiencies or blood concentrations above reference ranges, the results and advice are discussed by telephone with a sports physician.

An advantage of the Eetscore-FFQ as part of the NutriProfiel-Sport tool is that the overall diet quality of athletes can be assessed in a short time (15 min) with minimal burden. The disadvantage is that the Eetscore-FFQ is not a comprehensive questionnaire, and can only be used for a qualitative assessment of the diet rather than a quantitative assessment. Also, intake of supplements is not assessed with the Eetscore-FFQ. However, the Eetscore-FFQ was not developed to assess absolute nutrient intake, but as a screening tool to score the Dutch Healthy Diet index 2015 (DHD2015-index), and it is a valid tool for this purpose. If one is interested in a comprehensive assessment of dietary intake, other tools, such as a full-length FFQ or a 24-h dietary recall, should be used to capture a more detailed assessment.

### Strengths and limitations

A clear strength of our study is the availability of data from a large and diverse study population of athletes for the evaluation of the Eetscore-FFQ. Furthermore, we did not only evaluate NutriProfiel-Sport by means of an objective validation, we also included a subjective evaluation by asking athletes about their satisfaction with this tool. This is a strength of the study as appreciation and experienced usefulness are essential for acceptation.

A limitation of the study is that the number of athletes for the evaluation of dietary behaviour after using NutriProfiel-Sport and their satisfaction with this tool was rather low, and only twenty-nine of the fifty-five athletes who had indicated to be willing to participate in the study on the evaluation of NutriProfiel-Sport completed both the Eetscore-FFQ at baseline and at follow-up, and/or completed the satisfaction questionnaire. Another limitation is that micronutrient status in blood was not measured at follow-up, due to ethical and logistic reasons. Although diet quality had not improved at follow-up in the total population, some individual athletes had higher diet quality scores at follow-up, and it would have been interesting to investigate if this is also reflected in the micronutrient status.

## Conclusion

The Eetscore-FFQ as part of the NutriProfiel-Sport tool is a useful questionnaire for screening purposes of diet quality in athletes. In general, the coverage of nutrient intake of the Eetscore-FFQ in athletes was sufficient. Diet quality was high in our population of athletes, which may explain that diet quality did not improve after athletes received the dietary advice from the NutriProfiel-Sport tool. Nevertheless, a majority of the athletes reported the NutriProfiel-Sport results and advice as useful and valuable. Despite the relatively high diet quality, deviations from optimal micronutrient concentrations were observed, which underlines the need to monitor micronutrient status in athletes. NutriProfiel-Sport is a potential valuable and appreciated tool for this purpose.

## Supplementary material

The supplementary material for this article can be found at https://doi.org/10.1017/jns.2023.68.
